# Validity of facial skin analysis pore detection: A comparative analysis

**DOI:** 10.1111/jocd.16412

**Published:** 2024-05-31

**Authors:** Hannah Y. Gantz, Priscila Arellano Zameza, Mallory Zaino, Shirley P. Parraga, Jessica Q. Duong, Sarah L. Taylor, Steven R. Feldman

**Affiliations:** ^1^ Center for Dermatology Research, Department of Dermatology Wake Forest University School of Medicine Winston‐Salem North Carolina USA; ^2^ Department of Pathology Wake Forest University School of Medicine Winston‐Salem North Carolina USA; ^3^ Department of Social Sciences & Health Policy Wake Forest University School of Medicine Winston‐Salem North Carolina USA

## Abstract

**Background:**

Reliable, objective measures to assess facial characteristics would aid in the assessment of many dermatological treatments. Previous work utilized an iOS application‐based artificial intelligence (AI) tool compared to the “gold standard” computer‐based and a physician assessment on five skin metrics (*British Journal of Dermatology*, 2013, 169, 474). The AI tool had superior agreement for all skin metrics except pores and subsequently underwent an algorithm update for its pore detection system.

**Aims:**

This comparative analysis assessed the performance of the updated AI tool's pore scores across all Fitzpatrick skin phototypes to determine whether the AI tool more accurately represents a dermatologist's assessment of pores.

**Patients/Methods:**

Frontal facing photographs in uniform lighting conditions were taken of each participant. Percentile scores were generated by each of the four self‐learning models of the updated AI tool. The pore percentile scores generated by the original and updated AI tool were used to rate “worse” pores among participant pairs. These ratings were compared to pore assessments performed by a “gold‐standard” device and a board‐certified dermatologist.

**Results:**

Compared to the original pore detection tool and the computer‐based program, models A and D had the highest concordance with the physician's pore assessments for Fitzpatrick skin phototypes III‐IV and V‐VI, respectively.

**Conclusions:**

The AI tool's pores detection update was successful in its ability to accurately detect pores on all Fitzpatrick skin types, improving on the performance of the AI prior to the update. Responsibly developed AI tools that can accurately and reliably detect skin metrics across diverse Fitzpatrick skin types can facilitate dermatologic evaluation, individualize treatment, and determine treatment response.

The use of reliable, objective measures to assess facial characteristics has the potential to aid in the assessment of many dermatological treatments. Artificial intelligence (AI) using a deep neural network (DNN) algorithm to process external inputs, analyze patterns, and predict outputs may provide a means for doing so. Skin analysis applications using DNN technology can be easily downloaded onto handheld computing devices. Potentially widely available to the public, these applications can use live or stored user images to analyze and quantify common skin concerns like redness, wrinkles, and pores.

One skin analysis application utilized on an iPad Pro has been validated with a physician's assessment and a “gold standard” skin analysis device, showing that an application using DNN technology can provide data that is accurate and reliable across Fitzpatrick skin phototypes (FSP).^1^, ^2^ Trained on 70 000 faces from diverse ethnic backgrounds to detect 14 common skin concerns in seconds, this application generated percentile scores with a relatively high degree of agreement when compared to a dermatologists' assessment (Figure 1).^1^ The iOS application was also reliable under a variety of lighting conditions.^3^ The AI application outperformed a bulky computer‐based, “gold standard” skin analysis device in all metrics measured except for pores.^3^ When compared to the “gold standard” device and the physician's assessment, the AI tool had low agreement with the physician's assessment of pore quantity and quality in facial images, especially when assessing patients with FSP I‐IV.

**FIGURE 1:**
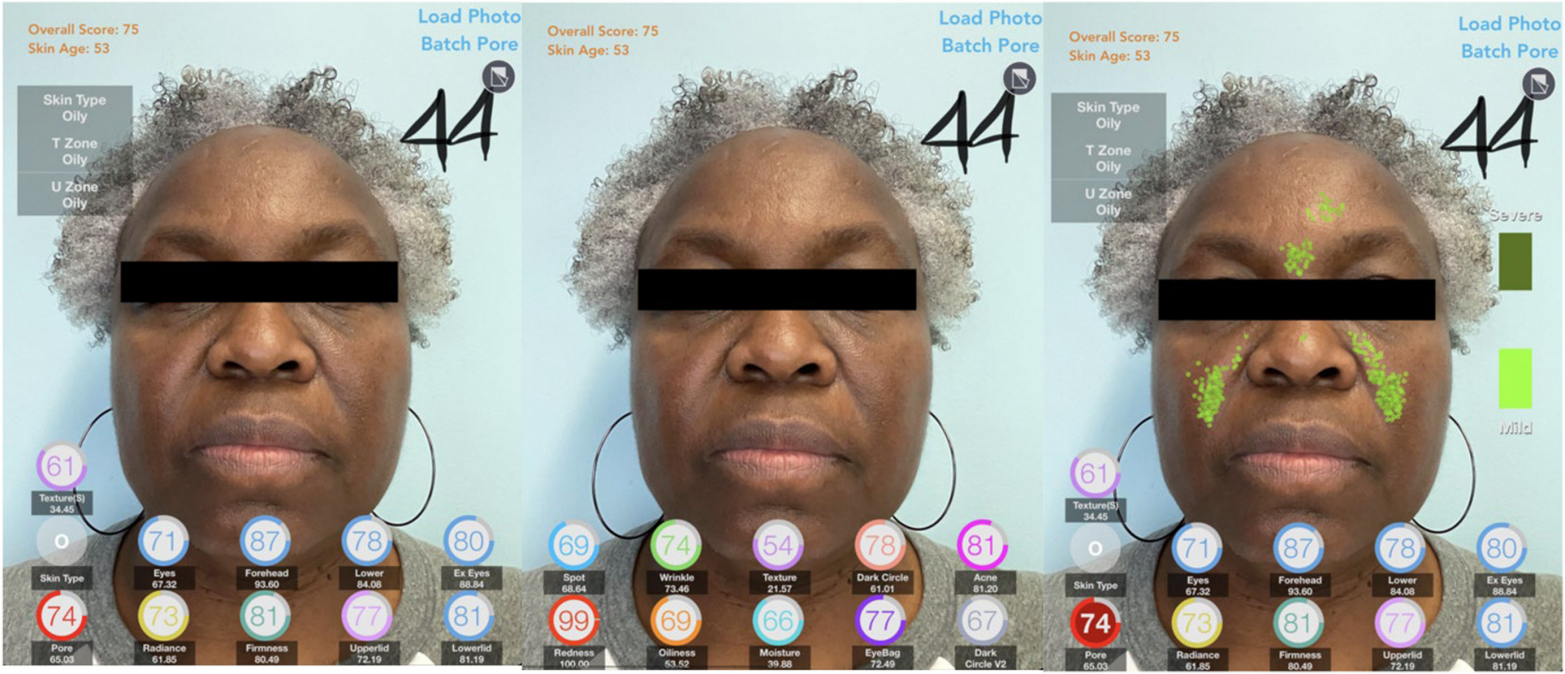
Example of a participant in Fitzpatrick Skin Phototype Group 3 with the iOS Application Technology. From left to right: two images demonstrating the skin metrics measured, and the pores overlay feature that highlights pores.

Pores refer to the openings for hair follicles and glands beneath the skin. These small channels play an important role in acne, one of the most common skin conditions in the United States, and rosacea.^4^ Enlarged, dense facial pores are a common cosmetic concern, with a variety of treatment modalities available to treat pores ranging from topicals to laser and chemical procedures.^5^ Having a device to measure pore size and density accurately and reliably would facilitate studies to evaluate pore treatments and to follow patients' progress. Updates in AI DNN pore detection algorithms may provide more accurate pore assessments.^6^ The iOS skin analysis application received an algorithm update to generate pore severity scores that more accurately represent a dermatologists' assessment of pore size, quantity, and distribution across all skin phototypes. The pores update included the addition of new DNN with deeper and larger layers to process external inputs (i.e., frontal facing images), analyze patterns, and predict outputs for the purpose of facial skin analysis. To represent a dermatologist's assessment more accurately, the DNN was trained on 70 000 images of faces representing diverse ethnic backgrounds (Figure 2). In this comparative analysis, we assess the performance of the AI tool's pore scores in all skin phototypes following a pores detection update. Enhanced performance and greater agreement with the physician's assessment may provide further validation for this AI tool compared to the “gold standard” device. This has implications for creating a more practical and accessible tool for measuring skin metrics across various skin types.

**FIGURE 2:**
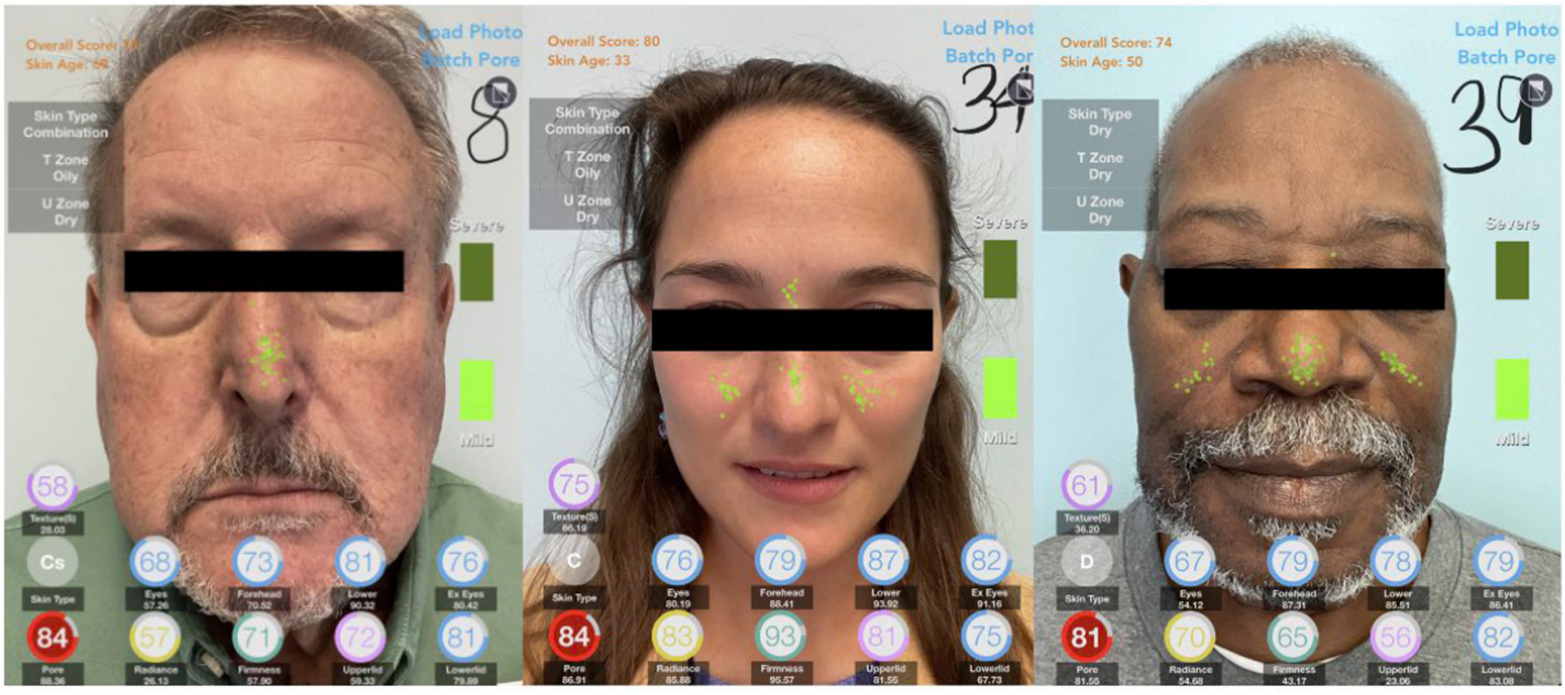
Example of Pores Feature across all Fitzpatrick Skin Phototypes (FSP). From left to right: FSP I‐II, FSP III‐IV, and FSP V‐VI).

We compared pore scores for both the original and updated tool (AI Skincare, Perfect Corporation) to a “gold standard” device (VISIA®, Canfield Scientific, Inc) and board‐certified dermatologist rater. Frontal facing photographs of adult participants were taken with an iPad Pro under uniform lighting conditions. Four self‐learning models (A, B, C, and D) of the updated AI tool generated percentile scores for each participant. With a minimum score of 0% and a maximum score of 100%, an individual's severity of pores is presented as a percentile (e.g., a score of 70 means that individual's severity of pores is better than 70% of same‐age individuals). A lower percentile score indicates larger, less desirable pores. The VISIA skin system also presents pore severity as a percentile score from 0% to 100%. Each model used different slightly parameters of the DNN to optimize pore detection. Images were numbered and paired consecutively (e.g., 1 vs. 2, 2 vs. 3, etc.). In each pair, the participant with the lowest percentile score for pores, indicative of “worse” pores, was recorded by each device. The physician rater looked at all paired images and characterized one participant in each pair as having “worse” pores. Paired participants with the same percentile score were excluded, as these pairs could not be directly compared to the physician's qualitative assessment. Concordance was determined by comparing rankings from the facial analysis systems to the physician's rankings; percent agreement was determined by dividing the number of concordant pairs by the number of pairs evaluated (Figure 3). Analyses were conducted using Excel.

**FIGURE 3:**
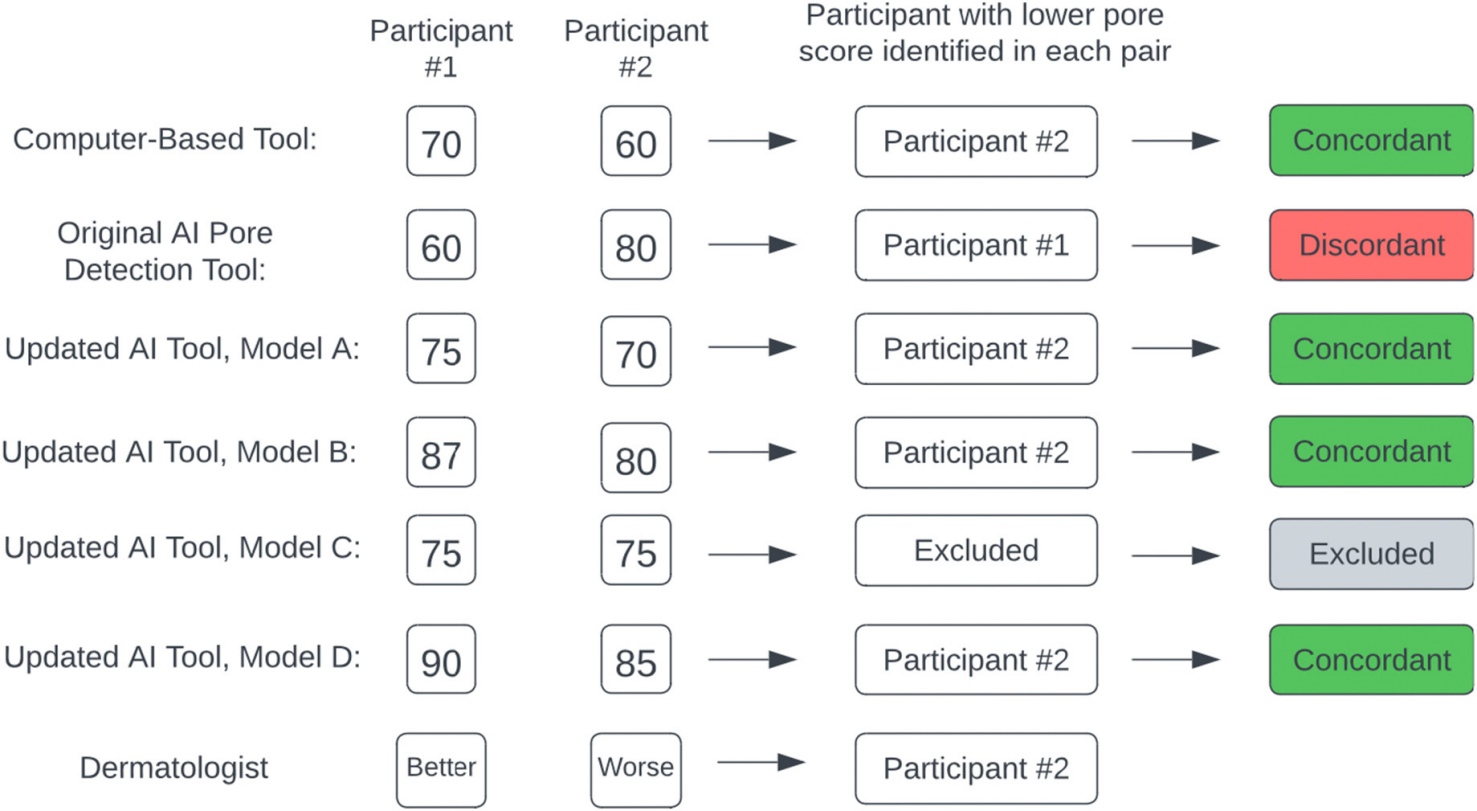
Illustration of study methods to determine concordant participant pairs. Numbers indicate the percentile score for pores, with a lower score indicating worse pores.

Fifty‐four participants (78% female) with FSP I‐II (*n* = 19), III‐IV (*n* = 18), and V‐VI (*n* = 17) were included to generate percentile scores that accurately represent a dermatologists' assessment of pore size, quantity, and distribution across all skin phototypes. After removing pairs with identical participant scores, 48, 49, 47, and 48 pairs for iOS models A, B, C, and D, respectively, were analyzed. Compared to the other skin analysis systems, models A and D had the highest concordance with the physician's pore assessments for FSP III‐IV and V‐VI, respectively. The updated Model D not only had the highest concordance score for Group 3 (66.7%), but also the highest total percent agreement (70.4%, Table 1). The “gold standard” device had highest concordance with the physician's pore assessments for FSP I‐II only.

**TABLE 1:** Percent agreement with physician score.

Fitzpatrick skin phototype (FSP) group	VISIA®	Original model	Model A	Model B	Model C	Model D
Group 1 (FSP I‐II)	**83.3%**	64.7%	55.6%	38.9%	76.5%	76.5%
Group 2 (FSP III‐IV)	66.7%	50.0%	**68.8%**	53.3%	53.3%	62.5%
Group 3 (FSP V‐VI)	43.8%	53.3%	60.0%	43.8%	53.3%	**66.7%**
Total	66.7%	57.1%	58.3%	46.3%	64.8%	**70.4%**

*Note*: VISIA® represents the “gold standard” device. The “Original Model” refers to the iOS application before the pores update. Models A–D represent the updated iOS application model following the pores update.

The values in bold are to emphasize the models with the highest concordance with the physician’s pore assesments compared to the other values.

The validated AI tool can detect and analyze 14 skin concerns. With the pores detection update, each metric assessed now performs equally across FSP skin types, closing the performance gap between lighter and darker skin tones.^3^ Compared to the original pore detection tool and the computer‐based system, the updated pore detection tool had the greatest percent agreement with the physician score for assessment of pore severity on darker skin tones. Future updates of the AI‐based IOS application can refine the algorithm in Model D, which had the best overall performance compared to other models and to the “gold standard” device. Basing its performance on the algorithm used in Model D only, the AI skin analysis tool can reliably assess skin metrics in a diverse patient population.

Limitations of this study include a single dermatologist assessment and the use of the FSP scale, a subjective classification, to stratify participants by skin tone without characterizing the range of skin tones that exist within phototypes with more melanin.^7^ The Monk Skin Tone Scale, for example, includes a wider range of skin tones and was adopted by Google to improve image diversity.^8^

The pores detection update was successful in its ability to accurately detect pores on all FSP skin types, improving on the performance of the AI prior to the update.^3^ The variation in the performance of the AI tool across skin types prior to the algorithm update is evidence of the need to assess the validity of AI measurement tools in patients with different skin types. Dermatologists can help set standards for AI facial skin analysis tools by encouraging the development of accurate and responsibly developed tools. This skin analysis tool follows the 2022 consensus guidelines from the International Skin Imaging Collaboration Artificial Intelligence Working Group.^9^ The consensus outlines 25 recommendations that serve as regulatory framework for automated dermatology algorithms by standardizing information including data (image types, technical acquisition details, external test set, etc.), technical assessment (performance measures, bias assessment, etc.), and potential impacts on the healthcare team and patients.^9^ The updated AI tool is compliant with 23 of the 25 recommendations, with the remaining two being non applicable to this tool's design, as it does not require histopathologic review for malignancy or references to other common diagnostic labels.

Although multiple skin analysis iOS applications are available for download, most exhibit performance limitations on darker skin tones.^7^ AI technology that generates reliable scores for a variety of skin metrics across skin types is a valuable research and clinical tool for dermatologists to track skin concerns, individualize treatment, and determine treatment response. AI‐based iOS applications can help eliminate interobserver variability. These tools are convenient, user friendly, and cost‐effective. AI tools like this iOS application can be used in both research and clinical settings to assess baseline skin metrics.^6^ Future research can assess the utility of AI‐based iOS applications to document treatment responses based on changes to percentile scores.

## AUTHOR CONTRIBUTIONS

Hannah Y. Gantz, BS, contributed to this work by assisting with the writing and editing of the original manuscript and formatting the manuscript for submission. Priscila Arellano Zameza, MS, contributed to this work by inputting data entry from the study, writing and editing the original manuscript, and formatting the manuscript for submission. Mallory Zaino, MD, and Sarah L. Taylor, MD, contributed by assisting with study design and edits to the original manuscript. Shirley P. Parraga, BS, and Jessica Q. Duong, BS, contributed to this manuscript by assisting with the editing and information gathering of the original and revised manuscripts. Steven R. Feldman, MD, PhD, contributed by serving as Principal Investigator for the study, overseeing study design, reviewing the paired images in this study, and editing and preparing the manuscript for submission.

## CONFLICT OF INTEREST STATEMENT

The Principal Investigator has received research, speaking and/or consulting support from AbbVie, Accordant, Almirall, Alvotech, Amgen, Arcutis, Arena, Argenx, Biocon, Boehringer Ingelheim, Bristol‐Myers Squibb, Dermavant, Eli Lilly and Company, Eurofins, Forte, Galderma, Helsinn, Janssen, Leo Pharma, Micreos, Mylan, Novartis, Ono, Ortho Dermatology, Pfizer, Regeneron, Samsung, Sanofi, Sun Pharma, UCB, Verrica, Voluntis, and vTv Therapeutics. He is founder and part owner of Causa Research and holds stock in Sensal Health. All other authors have no conflicts to disclose.

## ETHICS STATEMENT

This study was reviewed and approved by the Institutional Review Board of Wake Forest University (IRB#00085220).

## CONSENT TO PARTICIPATE STATEMENT

Written informed consent for the publication of de‐identifiable material was obtained by the authors from any adults participating in this study.

## Data Availability

Research data are not shared.
